# Smart Scheduling of Electric Vehicles Based on Reinforcement Learning

**DOI:** 10.3390/s22103718

**Published:** 2022-05-13

**Authors:** Andrei Viziteu, Daniel Furtună, Andrei Robu, Stelian Senocico, Petru Cioată, Marian Remus Baltariu, Constantin Filote, Maria Simona Răboacă

**Affiliations:** 1 Research and Development Department, ASSIST Software, Str. Tipografiei Nr. 1, 720043 Suceava, Romania; andrei.viziteu@assist.ro (A.V.); daniel.furtuna@assist.ro (D.F.); andrei.robu@assist.ro (A.R.); stelian.senocico@assist.ro (S.S.); petru.cioata@assist.ro (P.C.); remus.baltariu@assist.ro (M.R.B.); 2 Faculty of Electrical Engineering and Computer Science, Stefan cel Mare University of Suceava, Str. Universitatii Nr. 13, 720229 Suceava, Romania; filote@usm.ro; 3 National Research and Development Institute for Cryogenic and Isotopic Technologies—ICSI Rm. Valcea, 240050 Ramnicu Valcea, Romania

**Keywords:** smart scheduling, smart reservations, reinforcement learning, electric vehicle charging, electric vehicle charging management platform, DQN Reinforcement Learning algorithm

## Abstract

As the policies and regulations currently in place concentrate on environmental protection and greenhouse gas reduction, we are steadily witnessing a shift in the transportation industry towards electromobility. There are, though, several issues that need to be addressed to encourage the adoption of EVs on a larger scale, starting from enhancing the network interoperability and accessibility and removing the uncertainty associated with the availability of charging stations. Another issue is of particular interest for EV drivers travelling longer distances and is related to scheduling a recharging operation at the estimated time of arrival, without long queuing times. To this end, we propose a solution capable of addressing multiple EV charging scheduling issues, such as congestion management, scheduling a charging station in advance, and allowing EV drivers to plan optimized long trips using their EVs. The smart charging scheduling system we propose considers a variety of factors such as battery charge level, trip distance, nearby charging stations, other appointments, and average speed. Given the scarcity of data sets required to train the Reinforcement Learning algorithms, the novelty of the recommended solution lies in the scenario simulator, which generates the labelled datasets needed to train the algorithm. Based on the generated scenarios, we created and trained a neural network that uses a history of previous situations to identify the optimal charging station and time interval for recharging. The results are promising and for future work we are planning to train the DQN model using real-world data.

## 1. Introduction

### 1.1. Motivation

The last few decades have been marked by rapid technological advances that have resulted in significant positive changes in daily life, as well in an increase of pollution levels. To address the issue of pollution associated with the transportation industry, extensive technological research and development has been carried out in order to pave the way for electromobility worldwide.

As pointed out in *Electric Vehicles for Smarter Cities: The Future of Energy and Mobility,* issued by the World Economic Forum in January 2018 [1], to prevent congestion and pollution in the urban areas that have already been and will continue to be reshaped by demographic shifts, it is required to implement radical sustainable and secure mobility and energy solutions. The authors underline the fact the charging infrastructure deployment should be based on an anticipation of the long-term mobility transformation. Moreover, they consider that ensuring a reduction of range anxiety and developing smart charging technologies are key elements in the EV market approach, as they would contribute to the adoption of electro-mobility.

Wang et al. [2] emphasize the need for a scheduling approach to bridge the gap between EV charging needs and charging station supplies, as well as to deliver a favorable user experience that will stimulate EV adoption. Franke et al. [3] have analyzed the psychological barriers in adopting electric mobility, interfering with purchase intentions, focusing on EV range, and underlining that recharging opportunities and intuitive interfaces can contribute to the reduction of ambiguity and anxiety. According to a survey performed by the McKinsey Center for Future Mobility, the scarcity of fast chargers represents one of the main impediments to the widespread adoption of EVs, ranking third after price and driving range, but given the technological advancements that are steadily increasing range and the more attractive purchasing costs, charging related issues are bound to become the most significant barrier [4]. With more and more EVs on the road, available connectors will become difficult to find and, even though services such as ChargePoint or ChargeHub provide users with real-time information related to available charge points, the option to make reservations for a later date has not been implemented so far [5]. This aspect is particularly important for EV owners travelling long distances, as having the possibility to make advance reservations for their multiple charging operations along the road would enable them to plan a smooth journey, without the stress related to the uncertainty of finding an available socket once they reach the charging station.

In this context, we propose a solution that addresses precisely the aforementioned issues, reducing range anxiety among EV owners and removing the uncertainty associated with charge point availability by ensuring a smart scheduling of electric vehicles charging using Reinforcement Learning.

### 1.2. Related Work

Over the last several years, we have witnessed an ever-growing interest in Machine Learning in general, and one of its techniques—Reinforcement Learning (RL)—with implementations in a variety of fields. RL is designed as an optimization method involving an agent that learns from the interactions with its environment, based on the rewards received and that consequently adapts its behavior and decides upon the best action to execute in the future. Ultimately, the agent’s goal is to establish a policy that, when applied to action selection, generates the best reward over a long period [6]. The agent learns positive behavior, which is an important aspect of reinforcement learning. This means that it incrementally modifies or acquires new behaviors and skills. Furthermore, RL makes use of trial-and-error experience (as opposed to dynamic programming which implies full knowledge of the environment beforehand). As a result, the RL agent does not require complete knowledge or control of the environment and only needs to be able to interact with it and collect information [7].

The literature covers the use Reinforcement Learning techniques in optimizing the usage of the power grid to flatten the load curve and to avoid frequent voltage fluctuations, to reach peak reductions [8,9,10,11], to ensure the optimization of EMS (energy management systems), and to enable energy savings [12]. Moreover, researchers propose to use RL to learn household energy consumption, so as to optimally schedule EV charging, ensuring the reduction of energy expenditure, as well as a cost reduction [11,12,13]. Using Reinforcement Learning (RL) and Fuzzy Reasoning, Alfaverh et al. [14] propose a domestic energy management system, which successfully integrates the feedback provided by the user. The researchers employ the Q-learning strategy to optimally schedule household appliances and EV charging operation in terms of energy costs, without interfering with the user’s preferences.

In a series of scientific papers [15,16,17,18,19], researchers recommend using deep reinforcement learning to address the limitations of traditional model-based approaches that need a model to predict and optimize the scheduling process of EV charging. The authors propose to tackle the scheduling problem as a Markov Decision Process (MDP), to address the issues related to the randomness of traffic, of EVs arrival at the charging station and the fluctuation of energy rates that makes the optimization charging schedules difficult to achieve. Wan et al. [20] have established an MDP model of the EV charging–discharging control strategy, performing a series of simulations that yielded promising results related to cost reductions. Furthermore, the authors introduce an optimization mechanism for distributed real-time scheduling. Mhaisen et al. [21] also engage in a study designed to address the issue of EV charging and discharging scheduling operations in real-time, with the use of reinforcement learning, proposing a model-free RL technique able to determine a charging and discharging strategy on its own, as opposed to most of the scheduling techniques that strive to model the uncertainties associated with the existing unknown variables (real-time prices, EV behavior, energy demand) and plan correspondingly.

Volagianni et al. [11] proposed a customer model focusing on EV owners, simulating their driving, and charging behavior and creating driving profiles using data available from various public statistics, using Reinforcement Learning to gain insight into individual household consumption. The Smart Charging algorithm they proposed is also designed to ensure the stability of the energy network. The advantage of using model-free Reinforcement Learning consists of the fact that no prior knowledge is needed, overcoming the uncertainties inherent to the EV charging process.

To address congestion management, Rigas et al. [10] analyze the issue from both the perspective of the EV users and of the charging points, suggesting that congestion at the charging stations could be avoided by directing EVs towards various charging points and by distributing charging points’ locations along the routes. The authors studied artificial intelligence techniques for establishing energy-efficient routing, as well as for selecting charge points, analyzing, at the same time, the possibility to integrate EVs into the smart grid.

Tuchnitz et al. [22] propose a flexible and scalable charging strategy for EVs, using a Reinforcement Learning algorithm to establish a smart system for the coordination of an EV fleet charging process. Unlike optimization-based strategies, the proposed system does not require variables such as arrival and departure time and electricity consumption in advance, as the neural network is able to approximate the correct decision, according to the current parameters.

Ruzmetov et al. [23] identifies the lack of certainty of EV drivers to have an available charging point once they reach the charging station on their route as one of the major drawbacks in adopting electromobility. The authors introduce a platform meant to ensure a constant cooperation between the different involved entities: the energy suppliers, the charging stations and the EVs and EV users, proposing an optimization of the EVs’ scheduling and allocation to the charging stations. The destination set by the drivers, as well as the battery level, are taken into account when proposing a charging station, ensuring that this does not divert them from the route.

Qin and Zhang [24] conducted a theoretical study that enabled them to create a distributed scheduling protocol meant to reduce the waiting time, consisting of both the queuing time and the actual charging time, during a trip, along a highway. The reservations made by EV drivers for their next charging and the reservation adjustments are based on the minimum waiting time communicated by the charging stations, which periodically update this information, so as to enable drivers to make the optimal selection, in terms of waiting time.

Weerdt et al. [25], too, propose a solution to address the issue of congestions at the charging stations and long queuing times, in the form of an Intention Aware Routing System (IARS), which enables vehicles to reduce their travel time by taking into account the intentions of other vehicles. A central system is fed with probabilistic information about the intentions of vehicles in terms of estimated time of arrival at the charging station and, therefore, it can predict overcrowding and associated waiting times.

Furthermore, to address the needs of EV owners, while at the same time avoiding charging station congestions and power grid overload, Liu et al. [26] have conducted extensive research and proposed a reinforcement learning-based method for scheduling EV charging operations. To this end, the authors created a framework to enable communication between the charging stations and the EVs, and then determined a dynamic travel time model, as well as the EV charging navigation model. To further optimize the distribution of the resources of charging stations located in the area, they used reinforcement learning to enhance the charging scheduling model. Ma and Faye [27] also propose a solution to forecast the occupancy of charging stations, with the use of an LSTM neural network, a solution that has yielded promising results and on top of which smart scheduling strategies could be developed.

Shahriar et al. [28] provide a comprehensive overview of the application of various machine learning technologies to analyze and predict EV charging behavior, identifying the lack of publicly available datasets necessary for the training of ML models as one of the major drawbacks in the field. The authors also underline that available data are rather irrelevant, as they are specific to certain geographical areas, with traffic and EV users’ behavior particularities that cannot be applied in other locations.

Mnih et al. [29] explore recent breakthroughs in deep neural network training to create a deep Q-network that makes use of end-to-end RL to “learn successful policies directly from high-dimensional inputs”. The authors claim that, using DQN, they managed to create a first AI agent capable of achieving mastery in a wide variety of difficult tasks. This research, along with its predecessor, Playing Atari with Deep Reinforcement Learning [30], could be considered the cornerstone of DQN, demonstrating that in complex RL contexts, a convolutional neural network trained with the Q-learning algorithm is able to learn control policies from raw video data.

Among the techniques that enable DQN to overcome unstable learning, Experience Replay has a significant impact as it stores experiences such as state transitions, actions, and rewards, which constitute required data for Q-learning, and it creates mini-batches for updating the neural networks. Learning speed is increased, and the reuse of past transition prevents cataclysmic forgetting [31].

In their paper entitled Deep Reinforcement Learning Based Optimal Route and Charging Station Selection, Lee et al. [32] propose an algorithm designed to return the best possible route and charging stations, reducing the overall travel time, taking into account the dynamic character of traffic conditions, as well as unpredictable future charging requests. To determine the best policy for the selection of an EV charging station, a well-trained Deep Q Network (DQN) has been used. The authors point out that due to dimensionality, it is difficult to use Q-learning with the lookup table method for large-scale problems in real-world scenarios. They used DQN to approximate the optimal action–value function.

To contribute to the fuel efficiency of plug-in hybrid EVs, Chen et al. [33] propose a stochastic model predictive control strategy for energy management, based on Reinforcement Learning. Furthermore, the authors employ a Q-learning algorithm to set an RL controller used in the optimization process. Liu et al. [34] also suggest a smart charging strategy, based on an incremental Deep Reinforcement Learning system designed to simultaneously address both the issue of fast charging station selection and route planning, while taking into account user experience.

### 1.3. Contributions of This Paper

The purpose of this paper is to propose a solution capable of addressing multiple issues related to EV charging scheduling, such as congestion management, scheduling a charging station in advance, and enabling EV drivers to plan optimized long trips using EVs.

The novelty consists of the smart charging scheduling system that takes into account multiple parameters such as battery charge level, trip distance, available charging stations nearby, other appointments and the average speed.

Given the lack of datasets necessary to train the Machine Learning / Reinforcement Learning algorithms, the novelty of the proposed solution also resides in the scenario simulator that generates labelled datasets required to train the algorithm.

Our paper follows a similar approach to the studies performed in [32,33,34] covering, however, the following gaps:

- Our algorithm recommends the charging stations at the beginning of the trip, which can be positioned along various alternative routes to the destination point. This way, users have more options to choose from and they can ensure a smoother journey, with no long queues, by slightly adjusting their itinerary so that they pass by the optimal charging stations;
- The existing scientific articles in the field that studied the same concept using a Reinforcement Learning method employed a specific number of charging stations, meaning that introducing a new charging station in the grid would require the neural network to be retrained. We use the DQN to compute a score for each charging station in the grid, score that denotes the likelihood of selecting the current charging station given the car battery level and position, and the charging station’s reservations. This way we can increase the number of charging stations in the grid without having to retrain the neural network.

### 1.4. Methodology

The present article proposes to illustrate the technical details for the implementation of the DQN Reinforcement Learning algorithm (deep Q-networks) for the smart reservation of a charging point for an electric vehicle, as it has been developed within the framework of the Smart EVC project. It represents a continuation of the studies carried out in Design Patterns and Electric Vehicle Charging Software [35], an overview and performance evaluation of open charge point protocol from an electromobility concept perspective [36] and Electrical Vehicle Simulator for Charging Station in Mode 3 of IEC 61851-1 Standard [37].

**Figure 1:**
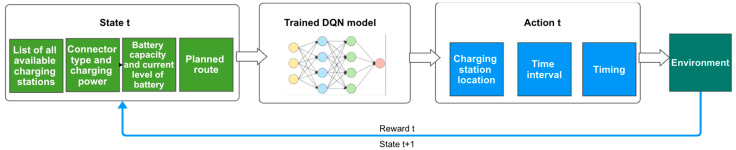
Study Methodology.

As illustrated in the diagram in Figure 1, the methodology employed while conducting the current study has as a starting point the parameters generated by the scenario simulator we had to create to compensate for the lack of relevant publicly available datasets. The datasets are required to train the neural network of the DQN model. The simulator feeds the algorithm with the data associated with a specific situation, based upon which the algorithm makes a decision. The scenario generator runs the simulation according to this decision and, subsequently, the algorithm is assigned a reward associated with the scenario, informing the algorithm of the correctness of its decision. Given the rewards it has received in each situation, the network is then trained to make better decisions in the future.

The smart scheduling of charging points for electric vehicles represents an issue that involves random variables. Given that it is difficult to create a dataset with the optimal solution for every potential situation, it cannot be addressed through Supervised Learning. For this reason, we have focused on a different class of Machine Learning algorithms, namely Reinforcement Learning.

The main goal of Reinforcement Learning algorithms is to identify the optimal solution to a problem, to maximize the reward function, in the long term. The RL agents learn by continuously interacting with the environment and observing the rewards in all circumstances. As we deal with an infinite number of possible situations, it is not possible to create a table to store the best solution associated with every situation. This challenge led us to the DQN algorithm, an RL algorithm using a function to decide the optimal solution in any situation. The DQN algorithm uses a neural network to decide upon the optimal solution.

### 1.5. Structure of the Paper

The remainder of this paper is structured as follows: after this introductory section, the charging station selection process based on Reinforcement Learning is described, with a presentation of the DQN algorithm and its training, of the communication protocol between the DQN and the proposed scenario generator, as well as the envisaged workflow. Section 3 covers the Smart EVC case study, followed by an analysis of the results achieved following the experiments based on the scenarios generated by the simulator. At the end of this publication, we have provided the conclusions and limitations of the current study, as well as envisaged future research directions.

## 2. Reinforcement Learning Based Charging Station Selection

In this section, we define the simulation environment and the RL algorithm we propose for smart scheduling of the electrical vehicles charging stations. We used an RL approach because of the scarcity of publicly available datasets that would enable the use of a supervised ML algorithm. Moreover, the RL approach is useful for handling uncertainty, which is characteristic to real-life situations. The advantage of this method is that the neural network employed by the DQN Reinforcement Learning algorithm uses a function to decide upon the optimal solution in any given situation, learning based on the trial-and-error principle.

### 2.1. The DQN Algorithm for Charging Station Selection

The DQN algorithm was written in Python based on the article “Human-level Control Trough Deep Reinforcement Learning”, published in 2015 [29].

#### 2.1.1. The Neural Network

The neural network was written using the Keras module from the TensorFlow library. To begin with, we opted for a simple neural architecture, which can be extended later, following the experiments. Because training this type of network requires extensive time resources, the neural network model is automatically saved and then loaded during the next iteration.

The Neural Network architecture of the DQN model consists of the following layers: a 7-neurons input layer, a hidden layer with 16 neurons and the output layer with one neuron. The model is compiled using the Adam optimizer and the Huber loss. The neural network’s weights are saved after each 100 iterations and the last checkpoint is loaded when we restart the app.

#### 2.1.2. The Replay Memory

For training purposes, we needed a history of the situations encountered thus far, as well as rewards associated with each decision made by the algorithm. To keep track of all scenarios, we created the Replay Memory class that allows us to store new scenarios.

The Replay Memory class stores two lists: the first one stores the scenarios without the reward and the second one saves the scenarios with their associated reward, assigned after the simulation of that scenario. When it receives the reward for a specific scenario, it is then moved from the first list to the second one, to avoid storing the same data in two locations. The training is performed only on scenarios from the second list, as we cannot train the DQN model on data without results.

A study [29] suggests a maximum replay length of 1,000,000. We reached the conclusion that this causes memory issues, and therefore we used a maximum length of 10,000. When the Replay Memory exceeds the 10,000 scenarios limit, the oldest items in the list are removed. The role of the experience replay is to enable the DQN model to learn from a more varied dataset. A small replay buffer size might lead to biased learning, meaning that the model will recall only the newest experience. Although in the long run it might still converge, the experience replay serves little to no purpose. On the other hand, a very large replay buffer leads to an increase in the training time, as older experiences might be reinforced.

When the DQN algorithm makes a decision, the simulator’s generated situations are stored. The simulator then runs based on the algorithm’s previous decisions and assigns a reward. This reward is automatically associated with the respective scenario, yielding a dataset that can be used to train the neural network.

#### 2.1.3. The Decision-Making Process

The DQN model has two types of decision-making process:

- Exploration—In this phase the decision-making process is mostly random, so that the model can explore more and discover what results are yielded by different decisions. This enables the agent to improve its knowledge about each action, which is usually beneficial in the long-term. In the beginning, the agent uses this random decision-making process and starts to gradually exploit its knowledge. By the time it reaches the 10,000th iteration, this exploration process probability reaches its lowest point, where it stays for the rest of the training time. To eliminate any misinterpretation, the fact that the number of iterations is equal to the size of the replay buffer is merely a coincidence and bears no relevance in the current study;
- Exploitation—In this phase the agent is exploiting its knowledge and opts for the greedy approach to get the most reward.

### 2.2. The Training of DQN

The neural network of the DQN algorithm is trained after every n decisions, n representing a hyperparameter of the DQN class. The consulted paper recommends starting a training session after every four algorithm decisions, which is the value used in the current implementation. The training of the neural network is performed based on 32 situations randomly selected from the Replay Memory.

Below, we have provided the steps involved in training the DQN model’s neural network:

1. Create a list of 32 random indices from 0 to the length of the Replay Memory;
2. Get a list of samples from the Replay Memory based on the indices list created at step 1;
3. Split the sample in state and reward lists;
4. Train the neural network for one epoch using the state and reward lists as input.

### 2.3. Communication Protocol between the DQN and the Scenario Generator

To enable communication with the scenario generator, a Python server has been implemented, using the FastApi library. The Endpoint recommending the charging station receives as body parameters information related to the current situation and returns the station’s ID and the time slot of the recommended reservation.

The “recommend_charging_station” endpoint is the main piece that connects the scenario generator with the DQN model. This endpoint expects as input the total distance of the trip, the battery capacity, the current level of the battery and a list of charging stations, each charging station with its own id, charging power and reservations. As for its output, it returns the id of the recommended charging station and the start and end points of the interval recommended for reservation.

When a request is made, the received data are processed so that it can be fed to the neural network. The processed data are then used to make a prediction for the charging station best suited to the current scenario. Before returning the data, it is processed again to create the interval of time recommended.

### 2.4. The Scenario Generator

The scenario generator simulator is written in JavaScript using the PixiJS library. An essential component of this simulator is the vehicle’s controller, this class being the main element that generates new scenarios and assesses the rewards assigned following a decision.

The car controller class has multiple properties such as the car’s speed, the current battery level, the battery capacity, and the consumption per KM. It also has a method that decides its next destination and it controls the direction it should take at each frame.

There are two other important classes employed by the scenario simulator. The first one defines the id, position and charging power of a charging station and the second one keeps track of all reservations with their charging station id and interval.

For the purposes of the current study, we have created an administration interface that enables us to create charging stations randomly positioned, as well as vehicles that start their journey from a random place and have a random destination. To this end, the simulator has created three interconnected cities, to allow for the simulation of the scenarios. The simulator communicates with the Python server through the Axios library.

**Figure 2:**
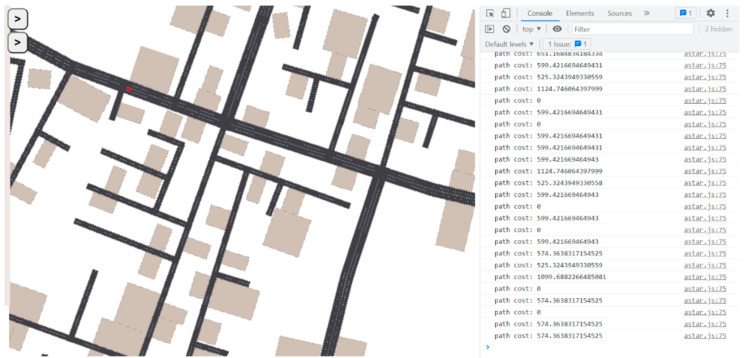
Simulator Interface.

The simulator’s interface is presented in Figure 2, below:

### 2.5. The DQN Algorithm and the Scenario Generator Workflow

**Figure 3:**
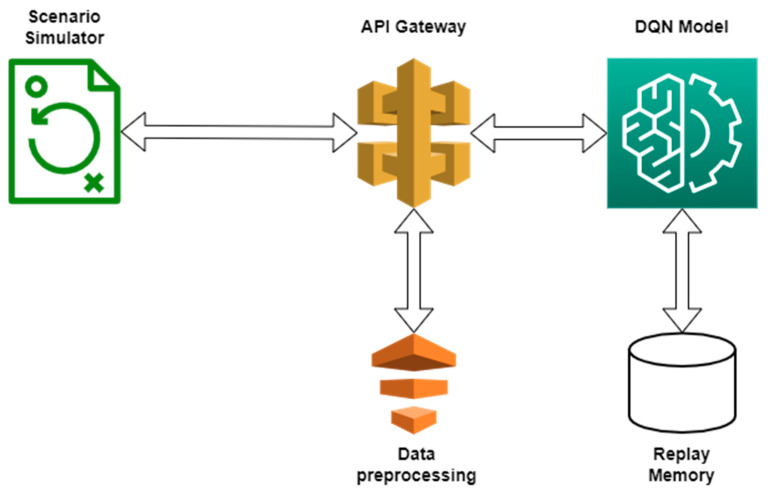
DQN Algorithm Workflow.

We have provided below, in Figure 3, a diagram summarizing the DQN algorithm workflow, along the scenario simulator.

The workflow presented in Figure 3 above can be summarized as follows:

1. A new situation is generated by the simulator and a request is made to the charging station recommendation endpoint, feeding in the information associated with the generated scenario;
2. The FastApi server receives the request and maps the data in the format required by the DQN algorithm. The processed data are then fed into the DQN algorithm;
3. The DQN algorithm makes a decision according to the current learning level or, with a certain probability, makes a random decision. The current scenario is then stored in the Replay Memory;
4. If there are enough entries in the Replay Memory, a training dataset is generated, which is then used to train the neural network of the DQN algorithm;
5. The DQN algorithm output is then passed back to the FastApi server, where another processing function is applied to map the data in a usable format;
6. The data are returned as a response to the request initiated by the scenario generator and the simulation runs based on the data received;
7. The value of the reward is established based on the simulation and it is then sent to the FastApi server, along with the scenario’s ID;
8. Through the FastApi server, the reward reaches the Replay Memory and the scenario in question is updated.

## 3. Case Study

The research activities described in the present paper have been carried out within the framework of the Smart EVC project, which aimed to create an intelligent charging station management platform based on Blockchain and Artificial Intelligence allowing for user—charging station interactions. Among other things, the mobile app is designed to enable users to plan a trip, a feature that is especially useful for longer routes.

The “Plan a Trip” feature has the role of scheduling the reservation of charging stations along a route. The user can reserve the charging station recommended by the ML model or can choose other ones along the way.

**Figure 4:**
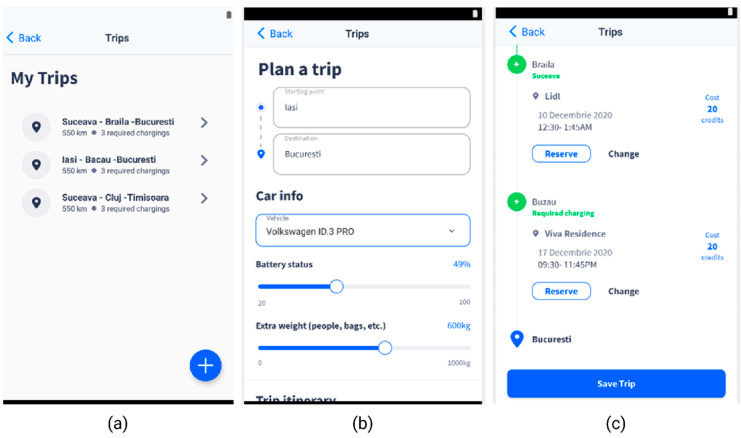
Smart EVC—Plan a trip section. (**a**)—EV trips list; (**b**)—Plan a trip feature, with details on the battery level and extra weight, based on which the necessary recharging operations are calculated; (**c**)—Proposed charging stations along the route, with reservation options.

When the user creates a new trip, the app prompts them to enter some relevant information (Figure 4b), which is critical for the DQN model’s accuracy and is related to the battery status and the vehicle type. This should be enough to assess the maximum battery capacity and the power consumption.

The history of the user’s trips (Figure 4a) provides data on previous trips that can be used as a training set to further refine the current model or train a new model from scratch.

**Figure 5:**
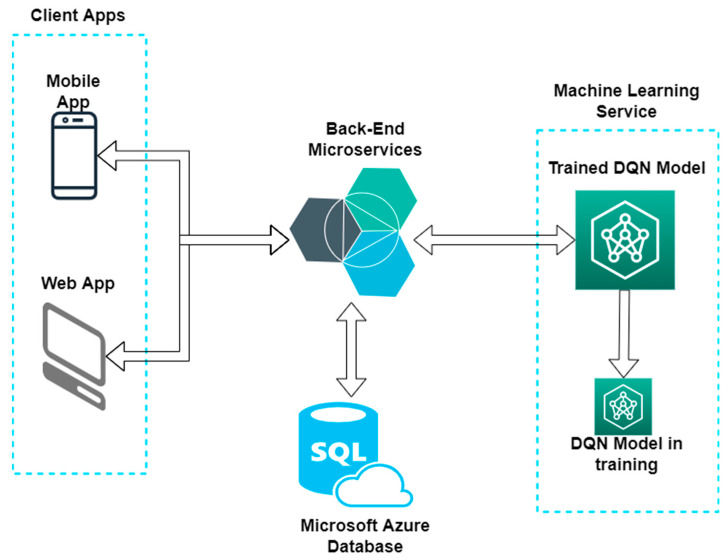
Architecture of the trained module.

As illustrated in the architecture in Figure 5, the charging stations selection system works as follows:

▪ The user sets up the starting and destination points for the trip in the mobile app, along with information about the vehicle and the battery status;
▪ The mobile app will call a Back-End endpoint with the data the user entered in the app;
▪ The Back-End will get from the database all the charging stations relevant for the data provided by the user and call the ML recommendation endpoint with the data received from database;
▪ The DQN model will predict the charging stations and the specific reservation time best suited for the data it gets from Back-End and sends them back as a response;
▪ The Back-End will send the response to the mobile app with the charging stations recommended by the DQN model;
▪ The mobile app will display the recommended charging stations to the user, with the option to create a reservation for the specified charging stations (Figure 4c).

## 4. Results and Discussion

Thus far, we have performed several experiments based on the scenarios generated by the simulator.

▪ Environment: Simulation of three interconnected cities, total distance of 25 km;
▪ Generated cars: 400 cars with a random initial position and random destination points. Once the destination point is reached, another destination point is generated. Each car sends requests to the DQN model whenever a new destination point is generated and if a charging station is returned, the vehicle’s route will be modified to reach the recommended charging station and recharge before continuing towards its original destination.

A request to the DQN model must send the necessary data to be fed into the model. When a request is received on the back-end, some additional data are retrieved from the database regarding the available charging stations and their reservations.

The data fed into the DQN algorithm are described below. The DQN model runs once for each charging station and time interval and returns a score. The charging station and time interval with the best score is then selected to be recommended to the user.

▪ The total distance between the current position of the car and the destination point;
▪ The capacity of the battery (in kW);
▪ The current battery level (in kW);
▪ The distance between the current position of the car and the charging station (in m);
▪ The length of the deviation from the original route and the changing station (in m);
▪ The charging power of the charging station per minute (in kW);
▪ The remaining time until the next charging slot (in minutes). A charging slot is a 30-min interval and is computed for the next 6 h starting from the current time.

After the simulation runs, the reward is appended to each decision the DQN model made. Examples of the inputs fed into the DQN model and the reward associated are presented in Table 1.

In these experiments we used a DQN with a simple neural network (a single hidden layer of 16 neurons). For this model we used the Adam optimizer with a learning rate of 0.001 and the Huber loss for a more stable training. The first 10,000 scenarios were epsilon-greedy, meaning that the model took more random decisions to explore the environment. After the first 10,000 scenarios, the probability of taking a random decision is set at 0.1, so most of the time we exploit what the model learned so far.

Every scenario is saved in the Replay Memory and, after the simulation is run for that specific scenario, the Replay Memory is updated with a specific reward. The scenarios saved in the Replay Memory are then used to further train the DQN model. The training is performed after every four scenarios with minibatches of size 32. The scenarios used for training are drawn at random from the Replay Memory.

**Figure 6:**
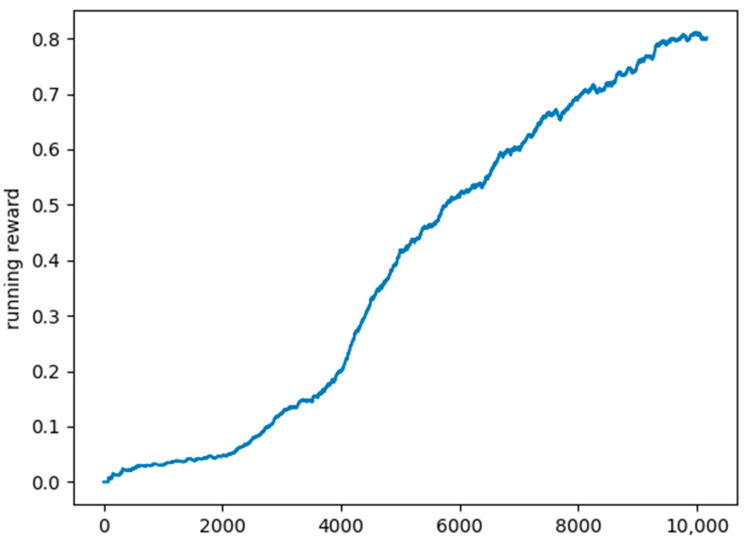
Plot of the mean running reward.

We chose as the evaluation metric the mean running reward over the last 1000 scenarios. Figure 6 shows a plot of the mean running reward in the exploration period. We can see that the model’s accuracy registers a steady growth in this period.

**Figure 7:**
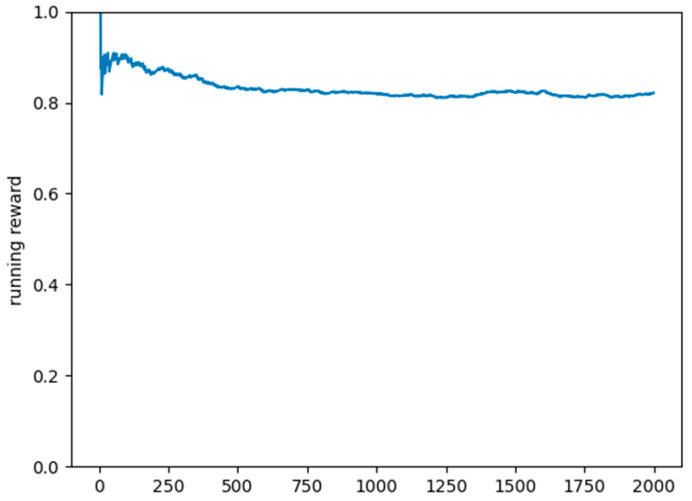
Plot of the mean reward on the testing set.

As a testing set, we used 2000 scenarios generated by the scenario simulator, where the DQN model only predicted the action to take, based on the previous training. In over 80% of the situations, the model predicted the correct charging station and the appropriate time slot. The plot in Figure 7 shows the mean running reward for the testing set.

## 5. Conclusions and Limitations

We have developed and trained a neural network that uses a history of the situations encountered thus far to identify the optimal charging station and time interval for recharging. Rewards are assigned to each decision made by the algorithm. With a lack of available datasets, a simulator that generates training data has been implemented, creating new scenarios.

Because the training data are generated by a simulator, they rarely resemble real-world data. If the DQN algorithm encounters situations that differ significantly from the scenarios in the training datasets, it may make incorrect decisions. The considerable training time of the neural network constitutes yet another constraint, as it needs to go through multiple situations and iterations to reach an optimal solution. Furthermore, the training time is extended because every scenario must be simulated before the algorithm is assigned a reward.

## 6. Future Research Directions

Going further, we are planning to train the DQN model using real-world data. These data can be retrieved from Google Maps through its Directions API. We can set up some fake charging stations to use as a reference, but these charging stations will use some real-world restrictions, such as being located within the boundaries of a city and (arguably) dispersed across the entire map.

With this setup, we can simulate scenarios with real-world distances, traffic, and car parameters. The model will then be trained using these scenarios and further refined when we start testing on real vehicles and charging stations. Training the DQN model this way will save us from having to train it from scratch with real data. We consider this a big win as training an algorithm from scratch implies a multitude of time-consuming scenarios.

## Figures and Tables

**Table 1:** Example of DQN model inputs and the associated rewards.

Total Distance (m)	Battery Capacity (KW)	Current Battery Level (KW)	Distance to the Charging Station (m)	Length of the Deviation (m)	Charging Power per Minute (KWm)	Time Remaining to the Charging Slot (min)	Reward
139	40	21	374	629	1	36	0
120	40	35	0	0	0	0	1
463	40	13	379	391	1	6	0.2
620	40	14	1130	1049	1	6	0.13
574	40	17	0	0	1	3	0.9
650	40	13	600	661	1	15	0.94
4637	40	20	4637	0	1	3	0.75
4587	40	17	5157	1171	1	51	0.89
868	40	22	0	0	1	36	1
6008	40	15	1020	360	1	0	0.97

## Data Availability

Not applicable.
